# Cultural Competence and Cultural Sensitivity Education in University Nursing Courses. A Scoping Review

**DOI:** 10.3389/fpsyg.2021.682920

**Published:** 2021-09-29

**Authors:** Cinzia Gradellini, Sagrario Gómez-Cantarino, Patricia Dominguez-Isabel, Brigida Molina-Gallego, Daniela Mecugni, María Idoia Ugarte-Gurrutxaga

**Affiliations:** ^1^Qualitative Research Unit, Azienda Unità Sanitaria Locale-IRCCS, Reggio Emilia, Italy; ^2^Reggio Emilia Nursing Course, University of Modena and Reggio Emilia, Modena, Italy; ^3^Faculty of Physiotherapy and Nursing, University of Castilla-La Mancha, Toledo, Spain; ^4^Research Group Nursing, Pain and Care (ENDOCU), University of Castilla-La Mancha, Toledo, Spain; ^5^Health Sciences Research Unit: Nursing (UICISA: E), The Nursing School of Coimbra (ESEnfC), Coimbra, Portugal; ^6^Hospital Emergency Unit, Toledo Hospital Complex (CHT), Castilla-La Mancha Health Service (SESCAM), Toledo, Spain; ^7^Reggio Emilia Nursing Course, University of Modena and Reggio Emilia, Modena, Italy; ^8^Azienda Unità Sanitaria Locale–IRCCS, Reggio Emilia, Italy

**Keywords:** nurses, education, cultural competence, cultural sensitivity, health

## Abstract

When assessing the fragility that characterizes the health of an immigrant person, a culturally competent transformation of the nurse–patient teaching-learning process is necessary. Therefore, it is considered essential to incorporate cultural competence and intercultural communication in higher nursing education.

**Objective:**

To determine the content and knowledge of cultural competence and intercultural communication offered in higher education in nursing courses.

**Design:**

The Campinha-Bacote model of cultural competence was used as the primary reference.

**Method:**

A scoping review was conducted about studies published in the period 2003 and 2020. The research was conducted between May and October 2020. More than a hundred documents (books, chapters, articles, conference proceedings) have been consulted.

**Results:**

Undergraduate nursing courses and postgraduate education move toward promoting cultural competence and sensitivity through teaching strategies.

**Conclusions:**

Teaching projects that combine multiple competencies are more effective, including teacher training. A predominant element is a need for continuous and transversal projects. University nursing education must adapt culturally competent curricula.

## Introduction

The incorporation into the European Higher Education Area (EHEA; Somoza et al., 2011), has meant changes of great importance in university teaching (Sánchez-Ojeda et al., 2018). This fact has led to significant legislative, professional, and social changes. Therefore, introducing a series of transformations at a structural and operational level has been promoted, directly impacting the skills and professional profiles of university education. This change process has given an increase to a teaching paradigm in which the student becomes the center of the entire educational process: this has led to a redefinition of both roles of teachers and students (Backes et al., 2011).

It is known that first-level nursing higher education is oriented to professional activities, so they need to work a lot on the students’ training (Backes et al., 2011; Gómez Cantarino et al., 2015). Successful completion of these undergraduate courses will give access to a Bachelor’s or Postgraduate degree, which appears in the Registry of Universities, Centers, and Titles (RUCT). Instead, second-level education (e.g., Master’s) is oriented to acquiring advanced training, as specialized or multidisciplinary topics. It also promotes initiation into research tasks (Gómez Cantarino et al., 2015).

University training in nursing follows a human, scientific, and teamwork perspective. They train students (in degree or Master’s) to identify, act, and evaluate the health needs of a target population. It also affects the health promotion and education for individuals, family, and community, considering their cultural environment (Backes et al., 2011; García et al., 2011). In this sense, Meleis (2010), a nurse theorist, indicates that nursing is considered a human and holistic science, with an orientation to care practice at any time and place, which begins with acquiring skills within the classroom (Cantarino et al., 2015). Considering the requested skills, fundamental for care at any place, and for any target population, the care is considered culturally competent when congruent to the people’s values and symbols influenced by the culture (Leininger, 1994).

The concept of cultural competence has its origin in the theories of intercultural nursing, specifically within the Leininger model, in the 1970s (Leininger, 1994; Leininger and McFarland, 2006). This model considers the analysis of the different cultures concerning: nursing, care practices, values and beliefs, concepts of health and disease. Its outcome guarantees effective and meaningful nursing care, in line with the cultural values and context (Ibidem).

It was only in the mid-1990s that this concept became more important. Even the American Academy of Nursing defined cultural competence, indicating that it includes a complete integration of knowledge, attitudes, and skills, facilitating intercultural communication and interactions between people (Leininger and McFarland, 2006). For this reason, United States began to incorporate cultural competence into nursing studies in the 1980s, specifically in San Francisco. In 1982, California introduced specific graduate studies in nursing (masters) to educate students on cultural competence. The literature suggests it is necessary to plan the students’ training to recognize their attitudes, enabling them to instill positivity in their relationship with patients in hospital and community care (González, 2008; Gómez Cantarino et al., 2015).

The term, cultural competence, also began to be more considered in the scientific community, thanks to the emergence of new theories, which are following described. For example, Purnell (with Tilki and Taylor) presents a model of cultural competencies and listening skills useful for health care professionals. It starts from the consciousness of the professional, and it considers four phases in mutual interaction: self-awareness, cultural identity, Attachment to inheritance and family assets, ethnocentrism (Purnell and Fenkl, 2019). Spector’s health traditions model is based on recognizing specific behaviors strongly related to the culture of origin. It analyzes how people manage their health concerning these. Giger and Davidhizar’s (2002) model of cross-cultural nursing describe six cultural phenomenal which need to be known by the health professionals in order to guarantee effective care: the communication barriers/problems; space/setting; the social organization of the context; the dedicated time; the environment control; the biological modification (structure of the body) (Karaburak et al., 2014). Last is the Campinha-Bacote cultural competence model, which interprets cultural competence as a dynamic process, and it requests health care providers to consider this competence a priority. The main assumptions are: (1) cultural competence is a process; (2) it consists of five main elements: cultural awareness, cultural knowledge, cultural skills, cultural encounter, and cultural desire; (3) within the groups there is more variation than across them; (4) health care providers’ cultural competence is strongly related to services providing culturally responsive care for ethnically diverse people (Campinha-Bacote, 2008, 2007, 2002).

In 2019, which corresponds to 3.5% of the world’s population, 272 million migrants traveled worldwide. The vast majority of these people arrive in a new country with precarious health, generally due to the conditions in which they made the trip (Guild et al., 2020). Indeed, the living conditions in the new country and the stress of acculturation can cause a rapid deterioration of the health capital; good health has been verified in most of the migrant population, based on a selection of people from the country of origin who are young and in good health (Tizzi et al., 2018). It is even found that the differences observed in the health problems of migrants, compared to the local population, are constitutional, cultural, or endemic in the countries of origin (for example, cervical cancer, female genital mutilation, tuberculosis). In addition, mental health problems are observed, often associated with migration routes coupled with living conditions in the host country (Sánchez-Ojeda et al., 2018; Tizzi et al., 2018).

It is important to note that the literature indicates an improvement of cultural competence in the higher nursing education educational offer. However, some common problems still arise in the teaching plans and training: (1) the lack of consensus on what should be taught; (2) the related timing; (3) the lack of standard references; (4) a limited and inconsistent formal evaluation of educational interventions (Spector, 2018). It is a shared opinion that university nursing courses (Undergraduate/Postgraduate) need to educate and prepare future professionals, providing them skills to face challenges and complexity generated by cultural diversity; ability to understand that care includes tolerance, respect, and critical self-reflection; ability to understand that values (related to culture but not only) are strongly involved in the therapeutic relationship.

Therefore, nursing courses are called to adopt curricula that support cultural competence to make future nurses able to promote social justice in care contexts. This concern is framed in what today is clearly defined as a standard of socio-political knowledge in nursing (Munn et al., 2018).

The objective of this scoping review is to analyze how undergraduate nursing programs and postgraduate education are promoting cultural competence and sensitivity in the learning programs.

## Materials and Methods

### Study Design

A scoping review has been processed to analyze if and how nursing courses’ *curricula* promote cultural competence and sensitivity. A vision of the focus of the documents is even acquired, which allows researchers an evaluation, synthesis, and criticism of the evidence inherent to the objective of the study (Siles González, 2005; Peters et al., 2015). As the primary conceptual model, we use the Process of Cultural Competence in the Delivery of Healthcare Services Model (PCCSS), proposed by Campinha-Bacote (2008, 2007, 2002). It is the most promising model to guide research since it can respond comprehensively and globally in all dimensions to the proposed objective. It is also the reference point for the analysis carried out with the designed strategy.

**FIGURE 1:**
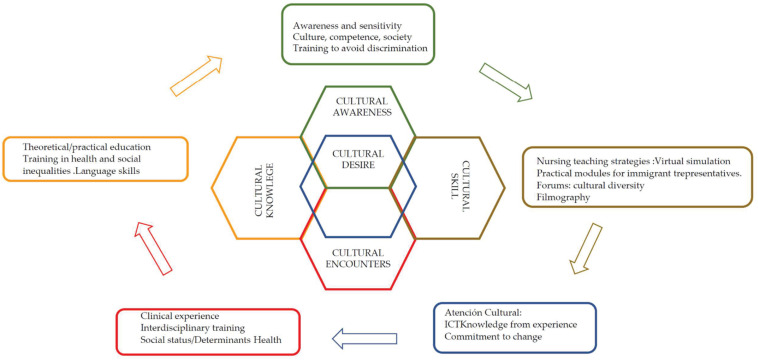
Campinha-Bacote (2002) model of cultural competence. Source: Authors’ own creation.

Through this model, culture provides the individual with a set of beliefs and values that define feelings of belonging and continuity. It also facilitates social integration and communication between members of a group (Campinha-Bacote, 2007). As previously mentioned, the PCCSS methodology is based on interpreting cultural competence as a continuous process. Students and health professionals strive to achieve skills with the different cultural groups of clients to serve (individual, family, community). Thus, cultural competence based on this model results from the integration of five concepts (1) Cultural awareness, which includes the exploration of one’s own cultural and professional background. In addition to the prejudices and stereotypes that are held toward people of different cultures. Therefore, it reviews cultural awareness and sensitivity through training (2) Cultural knowledge, where beliefs and values about health are integrated. To develop cultural competence within higher education studies in nursing (3) Cultural skills include identifying needs for care and adaptation to the context. Closely related to the development of didactic strategies in nursing university studies, (4) Cultural encounter promotes the need to involve the student and the health professional in inter-cultural immersions to prevent stereotypes. Use information and communication methodologies (5) Cultural desire, based on respecting differences and reinforcing similarities. It implies the will to learn on the part of the student–teacher (Campinha-Bacote, 2003) (Figure 1).

### Search Strategy

The scoping review process begins with an exploratory research question (Campinha-Bacote, 2003) to systematically synthesize existing knowledge (Colquhoun et al., 2014). In this research, we ask ourselves: “Are there specific didactic strategies to promote cultural sensitivity and competence in higher education in nursing?”

The model of cultural competence in the provision of health services (PCCSS) was applied. The researchers agreed on the eligibility criteria, containing information on cultural competence about nursing training and practice. The review also includes studies that contain teaching strategies and interventions. Studies on the cross-cultural theoretical/practical teaching-student experience were considered to consider the five elements proposed by the model.

The review includes books, book chapters, conference proceedings, and peer-reviewed articles. There are no restrictions regarding the country where the intervention was performed.

The exclusion criteria used are articles written in languages unknown to the researchers, articles without abstract, information that did not refer to the subject of the study.

The inclusion criteria are (1) articles from 2003 to 2020 (even if papers before this period are cited if they include theoretical models); (2) articles written in English, Spanish, Portuguese and Italian.

Several sources were consulted: (1) PubMed; (2) MedLine; (3) CINHAL; (4) Scopus; (5) Google Scholar. The terms MeSH and DeSH were used to carry out a more exhaustive and advanced investigation starting with the Boolean operator [AND], [Y], [OR]. In addition, combinations of words were used, where appropriate, to reflect the syntax and search rules common to the different databases.

The keywords used were the following: nursing, education; cultural competence; cultural sensitivity; Health; migrants, Social Determinants of Health.

After searching, eliminating, and selecting the articles, those selected were coded and identified as described in Table 1.

**TABLE 1:** Search strategy in databases.

Database	Search Strategy	Limits	Filters
PubMed	Enfermería AND educación Y Competencia Cultural AND Sensibilidad Cultural AND Salud AND Migrantes OR Determinantes Sociales AND Educación Y Conciencia cultural	Title Article English/ Spanish/Italian/ Portuguese	63 items filtered
MedLine			30 items filtered
CINHAL			24 items filtered
Scopus			25 items filtered
Google Scholar			172 items filtered

This scoping review took place between May and October 2020. This review aims to map the evidence supporting a particular research area and identify gaps in the existing evidence. This methodology does not intend to analyze the methodological quality of the included studies or find the best scientific evidence but rather to map the existing scientific evidence. Through the authors’ consensus (CG; SG-C; PD-I; DM; MIU-G), the publications were reviewed. A number of 105 documents were reached that met the requirements reflected in the inclusion and exclusion criteria. Researchers screened titles, abstracts, and full articles, independently and their bibliographic references manually to add them to the review. When discrepancies arose between investigators, the consensus was attempted, and, if not possible, an external reviewer resolved the conflict.

### Analysis of Data

The content analysis of the documentation was carried out from a qualitative perspective through an objective and systematic method (Peters et al., 2015). The steps that were carried out for the analysis consisted of (1) a thematic link; (2) a preliminary classification of documents based on content and organization criteria; and (3) a selection and extraction of the relevant information, according to the scope review criteria, in order to obtain results and conclusions (Campinha-Bacote, 2003; Siles González, 2004). The selected articles were analyzed from the point of view of the five concepts studied, which make up the PCCSS (Model of cultural competence): (1) cultural awareness; (2) cultural knowledge; (3) cultural skills; (4) cultural encounter; and (5) cultural desire. These concepts represent the meaning of working with cultural diversity, which encourages the caregiver to reflect on their cultural values (Campinha-Bacote, 2007, 2003). It is essential to specify that some studies covered more than one concept, so they have been analyzed in the category considered prevalent.

In this research about cultural competence, the researchers performed an inferential interpretation to extract and summarize the data. An attempt was made to know the reality already investigated and written regarding cultural competence in higher nursing education. The first and second authors (CG and SGC) carried out the general data extraction. While the third and fourth authors (PDI and BMJ) examined, the findings found. The fifth, and sixth authors (CG, DM, and MIUG) identified the standard thematic lines, which are included within the structures that make up the PCCSS (cultural awareness, cultural knowledge, cultural skills, cultural encounter, cultural desire). When a discrepancy in the studies’ inclusion appears, it has been resolved, searching for a consensus among the investigators.

The studies chosen for the analysis based on the degree of rigor were not excluded since the objective of the scoping review was to synthesize the results of the reviewed research to extrapolate a more excellent knowledge and vision of cultural competence to the scientific world (Crossetti, 2012; Table 2).

**TABLE 2:** Aplicación de la competencia cultural como proceso continuo a través del Modelo de J. Campinha-Bacote.

Theoretical model cultural competence	Concept treaty	Question-research	Document subject included	References
Campinha-Bacote, 2002	Cultural awareness	Are there preconceptions and prejudices on the part of health professionals toward other cultural groups?	Overview on cultural competence: ◯ Sensitivity ◯ Culture, competition, society ◯ Train and avoid discrimination	Clark et al., 2011; Lenburg, 1995; Lipson and Desantis, 2007; Meleis and Trangenstein, 1994; Meleis, 2010
				Chiarenza, 2012; Ingleby, 2012; Sealey et al., 2006; Purnell, 2005; Ibarra Mendoza and González, 2006
				Taylor et al., 2011; Maddalena, 2009; White, 2006; Atxotegui, 2000; Repo et al., 2017; Garneau, 2016; Meydanlioglu et al., 2015; Flood and Commendador, 2016; Bohman and Borglin, 2014; Jeffreys and Dogan, 2012; Bentley and Ellison, 2007; Allen, 2010
	Cultural knowledge	Is there enough knowledge about the vision that intercultural groups have of the world?	Cultural competence in cultural health training in Higher Education (Graduate/Postgraduate): ◯ Theories apply culture ◯ Virtual simulation ◯ Forums ◯ Filmography	Paul et al., 2014; Brathwaite, 2003; Foronda et al., 2016; Guzmán-Rosas, 2016; Montenery et al., 2013; Peek and Park, 2013; Noble et al., 2014; Carr and Knutson, 2015; Ahn, 2017; Lin et al., 2015; Riner, 2013; Callen and Lee, 2009; Upvall and Bost, 2007; Caffrey et al., 2005; Harkess and Kaddoura, 2016; Gradellini and Mecugni, 2015; Northam et al., 2015; Gallagher and Polanin, 2015; Truong et al., 2014; Calvillo et al., 2009; Morin, 1999; Aponte, 2012
				Gebru and Willman, 2010
				Sanner et al., 2010
				Watts et al., 2008; Cuellar et al., 2008; Gordon et al., 2016; Gradellini, 2013
	Cultural skills	Is there the ability to collect health data in a culturally sensitive way?	Other experiences: didactic strategies and adequate attention in Undergraduate/graduate: ◯ Theoretical/practical teaching: collecting cultural health data ◯ Training in social inequalities ◯ Multiprofessionalism and interculturality	Cenoz Iragui, 2004; Gradellini et al., 2012; Aponte, 2012; Sargent et al., 2005
	The cultural encounter	Do health professionals and students interact with people who belong to different cultures?	Development of cultural competence: ◯ Promotion of local, national and international care ◯ Stays in one’s own country adapting to the culture	Marshall, 2017; Hart et al., 2016; Siles Gonzalez et al., 2016; Ruddock and Turner, 2007; Bønløkke et al., 2018; Amerson and Livingston, 2014
	Cultural desire	Do you really want to be culturally competent?	The role of international mobility and the application of new technologies (TiC) ◯ Promotion of competent intercultural care ◯ Knowledge through experience	Koskinen et al., 2009; Upvall and Bost, 2007; Arbour et al., 2015; Hunter and Krantz, 2010; Giddens et al., 2012

## Results

### General Findings

**FIGURE 2:**
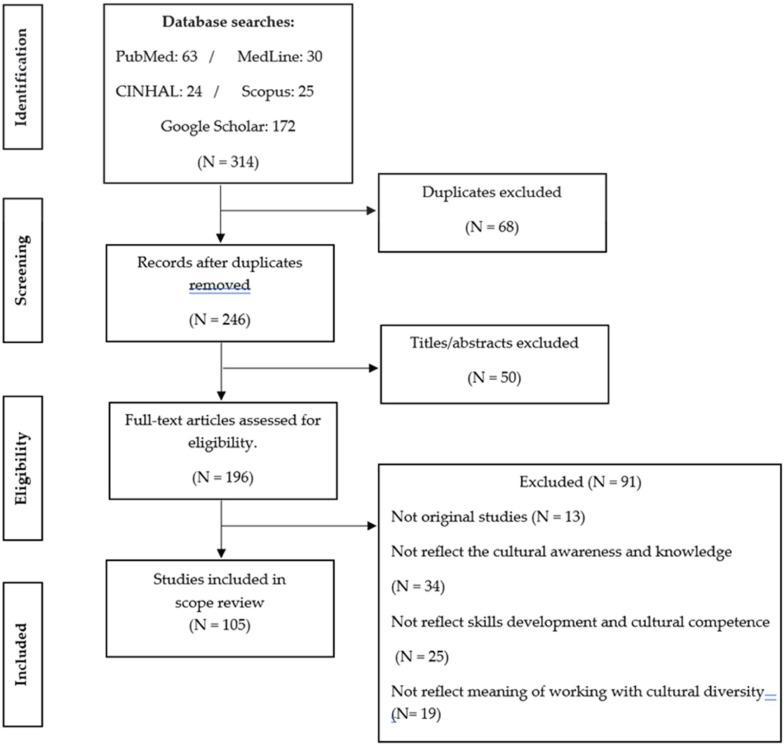
The flow of articles through the search process.

The search results flow through the scoping process is displayed in PRISMA. The initial search returned 314 articles, which were narrowed to 246 by eliminating duplicates. After reviewing the titles and abstracts of these studies, 51 additional studies were excluded. Of the remaining 196, 91 were eliminated for not being original studies since they did not include the subject of study (cultural competence/students), reflect cultural awareness and knowledge, consider educational skills, reflect on the meaning of working with cultural diversity. According To this, 105 studies were included in the scoping review (Figure 2).

### Cultural Awareness

Social diversity is constantly changing and linked to gender, age, religion, and belonging to a geographical and cultural environment. Health care professionals carry out their activity in a complex and very diverse socio-cultural environment, a consequence, among others, of the migratory processes that are taking place (Chiarenza, 2012; Ingleby, 2012). This interrelation between populations and cultures poses new challenges, requiring new resources and professional strategies to provide an effective and quality response (Taylor et al., 2011). Improving communication and understanding between the different actors (nursing-individual, family, community) that intervene in health processes has become a first-order need in the healthcare field (Maddalena, 2009). In this sense, cultural competence establishes an effective model to adapt the health services’ offer to guarantee citizens quality health care, adapted to their needs. Therefore, *cultural competence* can be defined as the complex integration of knowledge, attitudes, and skills that increases communication between different cultures (cross-cultural communication) and permits appropriate/effective interactions with others (Clark et al., 2011). Now, cultural awareness results from deep exploration (self-examination) of one’s own cultural and professional background, trying to manage the prejudices and stereotypes health care professionals could manifest toward people belonging to different cultures (Lenburg, 1995).

In North American contexts, to promote cultural competence, it has been developed specific teaching modules, such as the Culturally Competent Nursing Modules, proposed by the Office of Minority Health, and became a standard criterion of the Joint Commission for the accreditation of hospitals (White, 2006; Govere et al., 2016). Due to the increase of the immigrant population, with the consequent effects on the society, a similar module has been used in Spain, specifically in the city of Barcelona, in university training (Master), to promote the acquisition of cultural competence (Atxotegui, 2000). This reality has underlined the debate on intercultural processes and their influence on the Spanish health system (Sealey et al., 2006).

One of the strategies which could more than others promote cultural awareness is the experience to live the cultural difference. A Finnish study shows a generally moderate level of cultural competence in nursing students, with statistically significant differences between those who participated in multicultural nursing courses and exchange programs and students who are part of a minority or interact with other cultures (Repo et al., 2017).

Even knowledge of languages other than the mother tongue, which presupposes exposure to cultural difference, is an element that positively influences cultural sensitivity (Meydanlioglu et al., 2015; Garneau, 2016).

Although a review of the literature does not confirm the effectiveness of teachings of Transcultural Nursing if used as the only strategy to combat racist attitudes (Allen, 2010), several articles report validity of it (Bentley and Ellison, 2007; Jeffreys and Dogan, 2012; Bohman and Borglin, 2014; Flood and Commendador, 2016). Some indications emerge presenting strategies to integrate learning about prejudice and racism into the curriculum of nursing studies (Gordon et al., 2016). For example, the use of filmography as a tool for educational reflection is suggested, and the presentation and use of the Model of Cultural Attention of Madeleine Leininger (Gebru and Willman, 2003; Lancellotti, 2008).

Reflection on the concepts of equity in the health context, starting from the social condition and the determinants of health, has been considered a vital teaching methodology (Sargent et al., 2005).

In order to promote cultural awareness, a group of students participated in a teaching-learning project. This study was developed in collaboration with health programs planned to promote community literacy for refugees. The students referred to the immersion in the immigrant community as a positive experience (Harkess and Kaddoura, 2016). Most of these studies were carried out in Latin America (Honduras, Costa Rica, México, Dominican Repúblic, and Guatemala). One of the projects was conducted online, and the classes ran for 2 and 3 weeks. Two of the courses focused on language teaching, another on language skills. A fourth course was based on providing a quantitative vision by applying various scales such as the Cultural Knowledge Scale, the Cultural Diversity Questionnaire for Nursing Educators, and the Nursing Cultural Competence Scale (Sullivan, 2009). The didactic experiences’ evaluation shows students were culturally competent, and they achieved a greater awareness (Harkess and Kaddoura, 2016).

Using virtual contacts with different ethnic groups results in a good strategy for developing cultural awareness in care (Giddens et al., 2012). In general, stimulating a critical view of the concept of culture and cultural care is an important goal to develop empowerment, but it is often difficult to be achieved (Aponte, 2012).

An article suggests analyzing the students’ difficulties before acquiring cultural awareness to help them manage their management (Purnell, 2005). Considering that culturally competent nursing is sensitive to cultural differences, the attention to those difficulties could be an element of care (Meleis and Trangenstein, 1994; Meleis, 2010).

Promoting awareness about stereotypes and prejudices in order to avoid value judgments about other people without really knowing them is an important issue to be taught (Meleis, 2010; Purnell and Fenkl, 2019). Likewise, students must develop forums on cultural diversity to promote sensitivity toward other cultures (Sanner et al., 2010).

The current social dynamics make nursing care a permanent challenge since the values, beliefs, and practices of individuals, family, and community must be considered, first showing respect for the difference (Ibarra Mendoza and González, 2006). This process is not simple, and it requires a high level of awareness to have cared.

### Cultural Knowledge

Cultural knowledge for health care providers is fundamental to have specific skills and to provide culturally competent care. Knowledge of ethnic groups’ characteristics is fundamental: prevalent diseases (considering prevalence and incidence), the biological difference (e.g., in the metabolism of drugs (Paul et al., 2014), beliefs, and values related to health. These elements increase the cultural knowledge and let focused on the client’s point of view. To achieve the previously mentioned elements, the care providers must learn and use new cultural schemes (Brathwaite, 2003).

A scoping review highlights that nursing curricula introduced health-related issues and social inequalities to promote cultural awareness and competence in nursing students (Foronda et al., 2016; Guzmán-Rosas, 2016). A second review analyzes the commitment of university nursing departments to promote culturally competent care, considered imperative to promote the person’s centrality in nursing care at the local, national, and global levels (Montenery et al., 2013). Several studies claim that cultural competence can be developed by combining theoretical teaching of multicultural education (Peek and Park, 2013), based on scientific evidence (Noble et al., 2014), with experiences in the field. The knowledge could be used in contact with immigrants, refugees, and even vulnerable group populations (Caffrey et al., 2005; Kardong-Edgren et al., 2005; Sealey et al., 2006; Bentley and Ellison, 2007; Kardong-Edgren, 2007; Lipson and Desantis, 2007; Upvall and Bost, 2007; Callen and Lee, 2009; dit Dariel, 2009; Allen, 2010; Axtell et al., 2010; Jeffreys and Dogan, 2012; Riner, 2013; Bohman and Borglin, 2014; Carr and Knutson, 2015; Lin et al., 2015; Meydanlioglu et al., 2015; Flood and Commendador, 2016; Garneau, 2016; Ahn, 2017).

The collaboration of health professionals with immigrant communities’ spokespersons permit to define of some core elements of curricular studies, which could be subdivided into five areas: (1) self-awareness; (2) basic concepts (for example, culture and identity); (3) intercultural communication skills; (4) intercultural clinical skills; (5) promotion (Sullivan, 2009).

Two studies (a meta-analysis and a systematic review) intended to analyze the effectiveness of specific training projects to acquire cultural competence in nursing students. They declare that students training in cultural competence requires a broad explanation of procedures, practices, and potential problems, as well as a methodological rigor; the lack of agreement on a model which defines cultural competence is an obstacle to the development of the training itself (Truong et al., 2014; Gallagher and Polanin, 2015).

Let’s analyze teaching strategies to promote cultural competence in higher nursing studies (bachelor/master). Some courses promote didactic case management activities (Gebru and Willman, 2010), using the Problem-Based Learning (PBL) methodology (Gradellini and Mecugni, 2015). The use of 3D simulations of healthcare settings is even considered (Roberts et al., 2014). Also, in some developing countries (Everson et al., 2015), the use of specific programs to develop intercultural communication skills is promoted (Northam et al., 2015).

A Community Participation Research project (CBPR), in which the community is involved, promotes new opportunities for knowledge and nursing-individual relationship. It shows an increase of immigrant patients’ confidence in healthcare (Calvillo et al., 2009; Mathew et al., 2017).

The American Association of Nursing Colleges (AACN), as well as the European Federation of Nurses Associations (EFN), include the obligation to respect the biopsychosocial, cultural and spiritual integrity of people and to participate in the professional efforts to value life and quality of life, within their Code of Ethics. They have also made recommendations to promote elements of cultural competence (Calvillo et al., 2009). Even the United Nations Educational, Scientific and Cultural Organization (UNESCO) meeting in Paris dictated the necessary premises to include in the curriculum concerning cultural competence. In this sense, they identified five types of competencies: (1) the acquisition of knowledge about the social and cultural factors that influence care; (2) the use of evidence-based data to promote culturally competent care; (3) promoting safety and quality outcomes for people belonging to different ethnic groups; (4) the promotion of social justice, the health of vulnerable people and the elimination of health disparities; and (5) participation in the continuous development of competence, and cultural sensitivity (Morin, 1999; Calvillo et al., 2009; Sanner et al., 2010).

A combination of theoretical and practical activities is a suggested teaching strategy to promote cultural competence in undergraduate and graduate nursing students (Lipson and Desantis, 2007). Specific theoretical models related to culture and the acquisition of knowledge about the traditional medical practices used in the patient’s country of origin are presented in theoretical class, as researches’ results (Meleis, 2010; Purnell and Fenkl, 2019). The American Association of Colleges of Nursing (AACN) has developed an information package for educators (toolkit) that suggests culturally competent teaching strategies and care models (Morin, 1999; Calvillo et al., 2009; Sanner et al., 2010; Aponte, 2012).

The University of Pennsylvania has completed a process of change with the implementation of different activities developed through a working group made up of professors who are experts in cultural diversity. These are considered catalysts for change (Cuellar et al., 2008; Watts et al., 2008).

### Cultural Skills

If we consider the cultural skills, ones related to the patient’s assessment are considered fundamental. The Nursing Care Process (NCP) is a fundamental teaching in nursing studies theoretical and practical context, and the teacher must promote it. For what concerns its first stage, the nursing assessment, it is the neural center of the interview, that is why what to ask, when and how to do it, considering the specific of the professional cultural competence, should be taught with particular attention (Gallagher and Polanin, 2015). If we look at the patients’ medical records, where clinical paths are reported, it emerges that the patients’ perspectives and knowledge are conceived with the effectiveness of their care practices. This knowledge helps the nursing professional to plan the interventions that facilitate care, together with the individual. This approach enhances the patient’s satisfaction in the care (Cenoz Iragui, 2004).

A three-year project, funded by the European Community through the Erasmus Program working on cultural competence, has been presented. It involved ten professors and twenty students from four nursing courses from Italy (Reggio Emilia), Belgium (Antwerp), Finland (Turku and Seinajoki), working in a language other than the mother tongue, for 10 days. The course’s objectives were: (1) to experience being a stranger; (2) to work with students and teachers from other cultures; and (3) to reflect on what could influence intercultural care and which competencies should be acquired to care for patients in different phases life. All students participated actively in the concrete development of the program, in which experiential learning played a fundamental role. The evaluation of the project was carried out through the work of the students (content learning) and through two satisfaction questionnaires, which produced positive results (Gradellini et al., 2012).

A review of the most commonly used teaching strategies for cultural competence identifies clinical practice as the most effective element to develop awareness, knowledge, and security in students. It also advocates the development of standard cases and participation in projects that allow immersion in other cultures (Aponte, 2012).

An exciting project worked on cultural competence from a multidisciplinary perspective. Higher education students of the degree of nursing, educational sciences, and social work participated in a joint theoretical training about the process of Campinha-Bacote cultural competence. The practical didactic module was carried out using the Bennett Cultural Sensitivity Development Model. The project has been effective in developing personal awareness, acquiring knowledge about cultural differences, and ensuring culturally competent services (Campinha-Bacote, 2003). Other studies mention the Campinha-Bacote model of cultural competence as the most effective for developing specific competence in students (Campinha-Bacote, 2003; Aponte, 2012).

### The Cultural Encounter

International mobility programs allow students to immerse themselves in other cultures, enhancing cultural competence and awareness (Bohman and Borglin, 2014; Hart et al., 2016; Marshall, 2017). This effectiveness has been demonstrated through elaboration focused on the students’ experience, informative sessions, a briefing, and even through boarding diaries that reflect the experience (narrative approach) (Siles Gonzalez et al., 2016).

A revised study recognizes the efficacy of mobility using a phenomenological approach to Gadamerian hermeneutics, including open-mindedness, differences understanding, and fusion of horizons. Within this hermeneutical vision, horizons are characterized by: (1) range of vision between one culture and another; (2) changes made to adapt to another culture; (3) development of cultural sensitivity; and (4) individual growth. It is still a complex process, focused both on the student’s personal experience within an unknown context and a culture shock, without neglecting the decision-making that allows cultural adaptation. These are the actions to understand that sensitivity requires an openness to the multidimensional diversity of the new culture and demands an understanding of the value system as an element that influences the context of health and disease (Ruddock and Turner, 2007).

Even short periods of mobility (e.g., two weeks), accompanied by moments of guided reflection (tutor-learner), can increase: (1) cultural awareness; (2) a positive attitude toward the others; and (3) opportunity to compare differences and similarities (Bønløkke et al., 2018).

A descriptive study used reflective photography to evaluate the learning process of cultural competence. With this, the interaction of students immersed in a new cultural context during an international exchange program in Guatemala has been analyzed. The students were interviewed after reflecting on the images taken during the field activities. These portraits let to understand the experience (doing insight), in addition to generating awareness about a practice that has influenced cultural competence from a cognitive, affective, and practical point of view (Amerson and Livingston, 2014).

### Cultural Desire

Campinha-Bacote describes the health care providers’ cultural desire as motivation to engage in the process, creating cultural awareness, knowledge, skills, and encounter. Cultural humility also derives from this process, which refers to the will of the health professional to learn from the individual, family, and community the characteristics of their own culture (Campinha-Bacote, 2008, 2007, 2003, 2002).

Caring for individuals, families, or communities while respecting their own health culture implies formulating a culturally competent care plan. It means having the ability to consider the other’s point of view and recognize the human being’s subjectivity (Koskinen et al., 2009).

Regarding distance learning about cultural competence, some studies reviewed describe educational strategies that use Information and Communication Technologies (ICT). An issue that offers students from different cultures the opportunity to meet and develop the awareness, sensitivity, and respect necessary to provide culturally competent care (Upvall and Bost, 2007; Arbour et al., 2015). A semi-experimental study, applying distance training, carried out a pre-post evaluation applying the Assessing of Cultural Competence scale, and it demonstrated the effectiveness in terms of cultural diversity (Hunter and Krantz, 2010).

It should be taken into account that the use of a constructivist approach in the development of cultural competence shows that the knowledge of future nursing professionals is built from the lived experiences immersed in the culture of caring for people (Gebru and Willman, 2003; Hunter and Krantz, 2010). This approach could also be used to reduce inequalities in the health context, to promote social justice, and to promote critical reflection among nursing students after having developed practical activities (García et al., 2011; Montenery et al., 2013; Cantarino et al., 2015; Northam et al., 2015). Reflective conversations between teacher and student should be promoted to support students in giving means to what has been happened and felt to develop their own identity of care (Gradellini et al., 2012).

To analyze human responses in a context of cultural diversity, care professionals must alternate the activities programmed in the classroom with ones immersed in the care practice because such interventions implicitly carry desires, interests, motivations, expectations, and interpretations (Lipson and Desantis, 2007). Students feel comfortable and relaxing during the period in which this relationship develops is also the teacher’s responsibility since they are in charge of providing the resources in the acquisition of cultural skills, cultural encounters, and cultural desires.

## Discussion

An immersion in the EHEA has led to structural and operational transformations of all current university education. This process has concluded in a teaching paradigm in which the student is the center of the entire educational process (Cantarino et al., 2015). It is articulated in four main axes: (1) convergence in the structure and duration of the degrees (bachelors and masters’ degrees), to establish networks of European universities; (2) transferability, through the European Credit Transfer System (ECTS), adopted by all universities in the EHEA, to guarantee studies homogeneity and quality, as well as the academic and professional recognition of degrees throughout the European Union; (3) transferability of curriculum and professional qualification content and quality; and (4) mobility, to promotes competitiveness in the European labor market (Sánchez-Ojeda et al., 2018). Said axes suppose students and teachers cultural immersion at a national, European, and international level. This fact reinforces cultural diversity and even develops skills in acquiring general, transversal, and specific competencies throughout the students’ training period. This cultural immersion will contribute to the full development of the students, facilitating the “learning to do” and the “learning to be,” with a vision of the culture in which they are immersed (Backes et al., 2011; Gómez Cantarino et al., 2015).

These changes in higher education have generated modifications in the curricular plans of the Degree in Nursing. In them, work is being done to promote competence and cultural sensitivity through different teaching strategies (Bentley and Ellison, 2007; Jeffreys and Dogan, 2012; Bohman and Borglin, 2014; Flood and Commendador, 2016; Purnell and Fenkl, 2019). Some of these reforms are related to the development of cultural competence modules (Roberts et al., 2014; Everson et al., 2015; Gradellini and Mecugni, 2015); currently, specific postgraduate training in cultural diversity is being conducted promoted (Rozendo et al., 2017).

Thus, a specific university training is achieved and even with a specialized vision where fundamental concepts such as culture, stereotype, prejudice, and determinants of health are worked on (Sanner et al., 2010; Clark et al., 2011). On the other hand, the most practical activities within the undergraduate and postgraduate nursing degrees propose carrying out activities within the same setting where the care will be developed. The resolution of cases in the classroom is also promoted, and even the confrontation with vulnerable populations (Caffrey et al., 2005; Kardong-Edgren et al., 2005; Sealey et al., 2006; Upvall and Bost, 2007; Callen and Lee, 2009; dit Dariel, 2009; Gradellini et al., 2012; Riner, 2013; Lin et al., 2015; Ahn, 2017). In this sense, studies have been reviewed that focus teaching effort on increasing the understanding of students, future health professionals, toward the cultural framework of the patient. Although, indeed, the translation that this fact has in clinical practice is not specified (Hsieh et al., 2016).

Even in the development of clinical care, most patients continue to be cared for by health professionals and students in training, who emphasize biological or biopsychological aspects, making minimal effort to expand toward an integral dimension that considers cultural awareness counts (Renzaho et al., 2013; Hsieh et al., 2016).

Regarding cultural knowledge, most articles present a limited number of educational activities that emphasize social justice, cultural competence, security, and cultural promotion (Renzaho et al., 2013). Precisely, it has been verified that the activities identified concerning the educational offer determine contributions aimed at reducing inequalities in health and strategies to sensitize vulnerable populations (Lane et al., 2017).

As a clinical experience, the participation of students in cultural immersion projects, together with the use of clinical cases, is determined as a successful strategy in the acquisition of cultural competence (Sanner et al., 2010). Specific skills that could be recommended to enhance cultural competence have been proven. To give an example, the two-week teaching modules (Culturally Competent Nursing Modules), proposed by the United States Office of Minority Health, have been used as a Joint Commission standard criteria for the accreditation of hospitals and primary care centers (Allen, 2010; Noble et al., 2014).

Along these lines, the Liaison Committee on Medical Education (United States) established new accredited standards in 2000. These required students and health professionals to know the perceptions and explanatory models of different cultures’ people’s health and disease, and how these could vary. The response of patients to various symptoms or diseases (Sealey et al., 2006). The UK General Medical Council has also included cultural competence as one of the primary skills students must develop throughout their medical training (Horvat et al., 2014; Cantarino et al., 2015).

Health professionals reinforce the knowledge of the individual’s health culture and the traditional practices used by their patients, which appear essential to know all health-related practices. It has also been evaluated if these practices are oriented to promoting health, preventing the disease, or the cure. It is crucial that health professionals know and respect these practices to guarantee a confluence of traditional and conventional treatments (Siles González, 2005, 2004).

However, it is necessary to know if patients want a particular person (partner, family, friend, or relative) to be present during the care processes within cultural skills. Although it is true that the role that said person plays must be known. It has been proven that in the cases that act as translators of the native language, they can communicate errors in the transcription of health information (Kalischuk, 2014).

National, European, and international interuniversity mobility have an excellent learning impact since it encourages fieldwork in cultural contexts different from those learned (Peek and Park, 2013; Bohman and Borglin, 2014; Carr and Knutson, 2015). Although this situation indeed generates exponential learning of the new culture in future professionals, one must be cautious in believing that this situation conditions the knowledge of an entire community. Beliefs or practices held by a group cannot be generalized; within each group, there might be intra-group variation. A phenomenological study of Caucasian undergraduate nursing sciences teachers, who work on cultural differences, highlighted the need to ensure continuity in specific learning topics and promote relational exchange (Beach et al., 2005; Amerson and Livingston, 2014).

A point to consider is the personal attitude and sensitivity to specific issues of the target population of care due to personal experience (Guild et al., 2020). Following this line, it would be very positive to incorporate cross-cultural thinking into the nursing training curriculum, which induces awareness of humans (Garneau, 2016; Repo et al., 2017). The situation that leads to not reducing the human being to the lowest part of himself, on the contrary, the multiple aspects that each being brings in the person’s condition will be discovered (Charles et al., 2014). However, it is true that graduate students, in general, show a higher disposition when it comes to acquiring knowledge about other cultures (Watts et al., 2008; Sanner et al., 2010; Gordon et al., 2016).

Even the acceptance and respect for cultural differences, the sensitivity to understand how these differences influence relationships with people, and the ability to offer strategies that improve cultural encounters, are essential requirements for cross-cultural care in nursing to be consolidated.

## Conclusion

From this scoping review, the importance of defining a shared national, European and international curriculum in higher education on specific topics related to general, transversal, and specific competencies emerged clearly. Among these, the development of cultural competence stands out, and the need to define the actual effectiveness of educational strategies related to it.

Both the combination of different educational skills and the training of expert teachers should be considered critical points in the teaching of higher education in nursing. Many published experiences confirm the need to improve the acquisition of cultural competence in nursing students as future promoters of the right to health.

The described educational’s proposals underline common hindering factors for its development. The lack of knowledge about minority groups by health personnel is striking.

At the international level, the World Health Organization (WHO) recommends, since 1946, the highest level of health standards as a fundamental right of every human being. Therefore, equal treatment, access to health care, and respect for all people are a prerequisite for culturally competent care, where the person’s integrity is guaranteed.

*Nursing* is a science that is based on care and respect for individual differences. Therefore, it is unquestionable that the progress of this care and respect for the individual, family, and community is the first requirement to achieve culturally competent care, essential to guarantee the integrity of the person.

## Author Contributions

CG and SG-C contributed to conception and design of the study. PD-I, B-MG, DM, and MIU-G performed the collection, organization, and analysis of data. CG and SG-C wrote the first draft of the manuscript and the sections of the manuscript. All authors contributed to manuscript revision, read, and approved the submitted version.

## Conflict of Interest

The authors declare that the research was conducted in the absence of any commercial or financial relationships that could be construed as a potential conflict of interest.

## Publisher’s Note

All claims expressed in this article are solely those of the authors and do not necessarily represent those of their affiliated organizations, or those of the publisher, the editors and the reviewers. Any product that may be evaluated in this article, or claim that may be made by its manufacturer, is not guaranteed or endorsed by the publisher.

## Abbreviations

EHEA: European Higher Education Area
RUCT: Registry of Universities, Centers and Titles
PCCSS: Model of Cultural Competence in the Provision of Health Services Campinha-Bacote
NCP: Nursing Care Process
PBL: Problem-Based Learning
ICT: Information and Communication Technologies
ECTS: Credit Transfer System
WHO: World Health Organization.
